# Supplementary material for: The causal relationship between the human gut microbiota and pyogenic arthritis: a Mendelian randomization study
Source: Front Cell Infect Microbiol. 2024 Nov 26;14:1452480. doi: 10.3389/fcimb.2024.1452480 (PMC11629706; doi:10.3389/fcimb.2024.1452480)
Supplement: Supplementary file 1 [file DataSheet1.zip › mendelian test/ebi-a-GCST90027538.csv_forest.pdf]

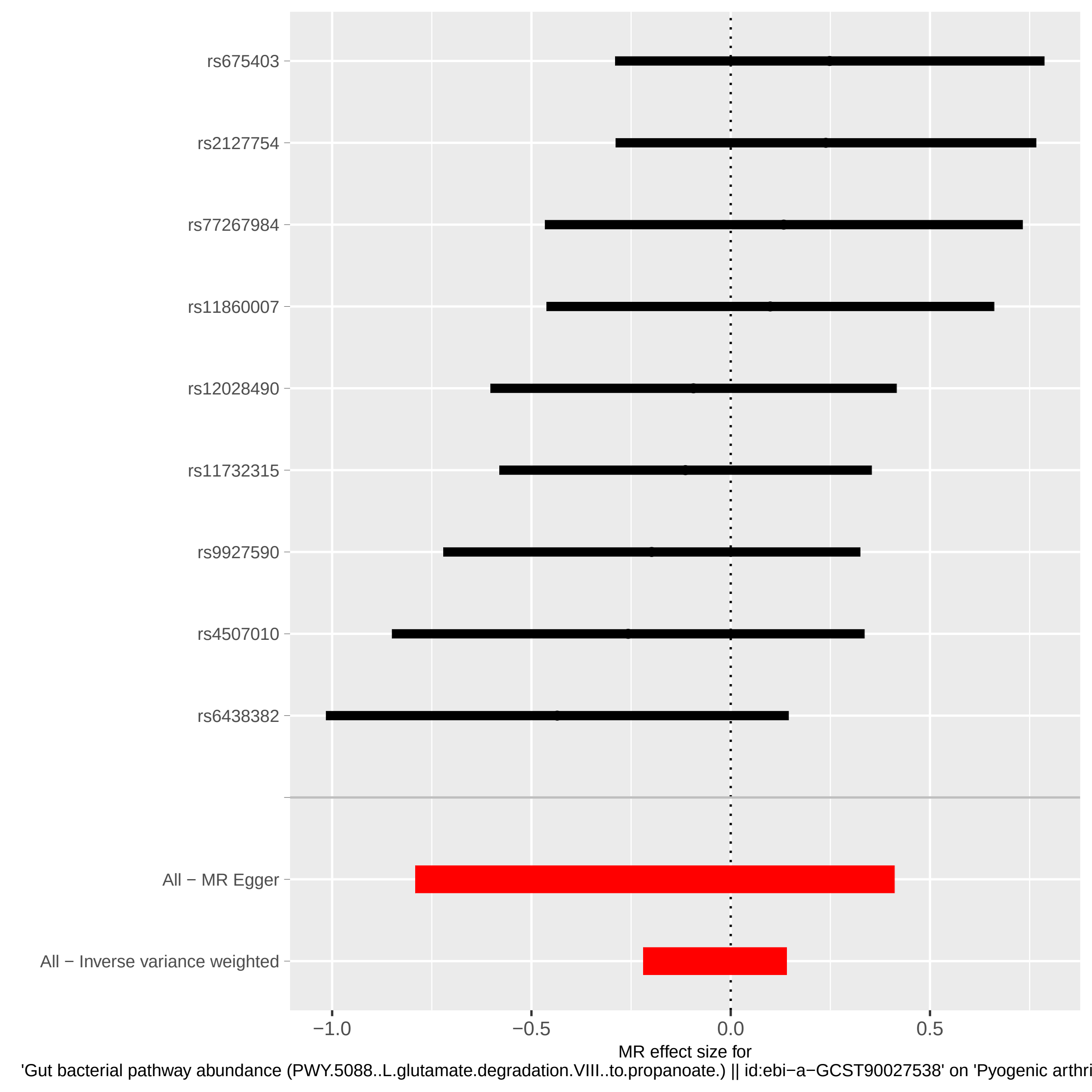
rs675403
rs2127754
rs77267984
rs11860007
rs12028490
rs11732315
rs9927590
rs4507010
rs6438382
All − MR Egger
All − Inverse variance weighted
−1.0
−0.5
0.0
0.5
MR effect size for
'Gut bacterial pathway abundance (PWY.5088..L.glutamate.degradation.VIII..to.propanoate.) || id:ebi−a−GCST90027538' on 'Pyogenic arthri
